# Iodised salt and iodine supplements for prenatal and postnatal growth: a rapid scoping of existing systematic reviews

**DOI:** 10.1186/s12937-015-0079-z

**Published:** 2015-09-02

**Authors:** Jessica Farebrother, Celeste E. Naude, Liesl Nicol, Maria Andersson, Michael B. Zimmermann

**Affiliations:** 1 Human Nutrition Laboratory, Institute of Food, Nutrition, and Health, ETH Zurich, LFV D 11.2, Schmelzbergstrasse 7, 8092 Zurich, Switzerland; 2 Centre for Evidence-Based Health Care, Faculty of Medicine and Health Sciences, Stellenbosch University, Stellenbosch, South Africa

## Abstract

**Background:**

Iodine deficiency can adversely affect child development including stunted growth. However, the effect of iodine supplementation or fortification on prenatal and postnatal growth in children (<18 years) is unclear. We identified the potential need for a systematic review to contribute to the evidence base in this area. To avoid duplication and inform the need for a new systematic review and its protocol, we undertook a rapid scoping review of existing systematic reviews investigating the effect of iodised salt and iodine supplements on growth and other iodine-related outcomes.

**Methods:**

We searched TRIP and Epistemokinos (latest search date 15 December 2014). All English language systematic reviews reporting on the effect of iodine supplementation or fortification in any form, dose or regimen on any iodine-related health outcomes (including but not limited to growth) were included. Eligible systematic reviews could include experimental or observational studies in pregnant or lactating women or children to age 18. We tabulated the extracted data to capture the scope of questions addressed, including: author, publication year, most recent search date, participants, pre-specified treatment/exposure and comparator, pre-specified outcomes, outcomes relevant to our question and number and type of studies included. Methodological quality of included reviews was assessed using AMSTAR.

**Results:**

Nine hundred and seventy-six records were screened and 10 reviews included. Most studies were of moderate methodological quality. Outcomes included assessments of thyroid function, iodine deficiency disorders, mental development and growth. Populations studied included pregnant women, preterm infants and children into adulthood. Most reviews looked at direct iodine supplementation or fortification, though some reviews considered iodine status, including the relationship between iodine intake and iodine biomarkers. Although five reviews pre-specified inclusion of growth outcomes, none provided synthesised evidence on the effects of iodine supplementation or fortification on prenatal and postnatal somatic growth.

**Conclusions:**

Our rapid scoping review demonstrates a gap in the evidence base with no existing, up-to-date systematic reviews on the effects of all forms of iodine supplementation/fortification in all of the relevant population groups on relevant growth and growth-related outcomes. A new systematic review examining this question will assist in addressing this gap.

## Introduction

Iodine deficiency has been identified as one of the key preventable factors that can adversely affect child development [1], and is one of the most widespread micronutrient deficiencies worldwide [2]. It can result in a number of developmental and functional abnormalities, the spectrum of which is referred to as the Iodine Deficiency Disorders (IDD) [3, 4].

As an essential micronutrient and component of the thyroid hormones, which regulate growth and development from conception to adulthood, iodine plays a major role in normal physical growth and development until well after birth [5]. The synthesis of the thyroid hormones is impaired when dietary iodine requirements are not met. In the first trimester of pregnancy, thyroid hormone from the mother crosses the placenta to fulfil the needs of the foetus [6]. It is well established that a deficiency in iodine during the entire pregnancy may cause thyroid dysfunction and have irreversible adverse effects on child development if moderate or severe [7–9]. Indeed, during the entire growth period thyroid hormone promotes the growth and development of peripheral tissues and the skeleton [5]. Hypothyroidism induced by iodine deficiency can thus have a negative impact on growth and development at all stages of the human growth cycle [7], and can lead to stunted growth if not addressed [10, 11].

In 2012, the World Health Organization (WHO) specified six targets for the 2025 Global Nutrition Agenda [12, 13]. The first was to address stunting. The specific target: a 40 % reduction in the prevalence of stunting in children under 5 years by 2025. Addressing stunting has thus become an urgent priority, and there is increased interest and need to find effective nutrition interventions to target this global burden. Stunting in childhood is associated with both short and long-term consequences, such as decreased cognitive function, slowed motor and language development, a decreased performance at school and a lowered learning capacity [14–17]. Though the prevalence of stunting is decreasing [18], recent global estimates from the United Nations Children’s Fund (UNICEF), WHO and the World Bank stated that in 2013, 161 million children under 5 years were still stunted [19]. Despite a continued decline, if current trends continue the WHO predict that in 2025, 127 million children under 5 years around the world will remain affected by stunted growth [13].

Within the design of health promotion packages for children, it is commonly overlooked that iodine deficiency has adverse effects on growth and correction of iodine deficiency is frequently not considered an important contributor in promoting optimal child growth and potentially reducing the risk of stunting. The most effective means to assure adequate population iodine nutrition and prevent IDD is universal salt iodization [4, 20, 21]. Where the coverage of iodised salt is incomplete, daily iodine supplementation is recommended to pregnant and lactating women and infants [4]. Doses of iodised oil given once or at repeated intervals may also be an effective intervention for vulnerable groups until an effective iodised salt programme can be implemented [4]. Iodine can also be provided via regular consumption of micronutrient powders, such as those distributed by UNICEF and World Food Program (WFP) programmes [22] and through fortification of other foods such as bread, which has been previously shown to have an impact on the iodine status of school-age children [23, 24].

Despite the clear evidence supporting universal salt iodisation for the prevention of IDD, there is a lack of clarity on the effects of iodine-related interventions on somatic growth and risk of stunting. This information is key in light of the recent spotlight on finding solutions for the global stunting burden. To address this gap, we formulated the following research question: “What are the effects of iodised salt, iodised oil or iodine supplements compared to placebo or no iodine intervention on prenatal and postnatal somatic growth of the foetus, infant and child?” We identified the potential need for a systematic review to tackle this question.

Synthesis and evaluation of all available relevant research would inform and strengthen the evidence base in this area. The current best evidence should inform decisions on health interventions and guidelines, particularly in resource-poor settings. Systematic reviews of the effectiveness of interventions, using explicit methods to reduce bias, have the potential to inform decision-makers as to which interventions to implement, modify or withdraw from health care [25]. While systematic reviews should be used to inform interventions and guidelines, it is not always necessary to do new reviews. If up-to-date, relevant and high quality systematic reviews exist, these should be used. Updating existing reviews, if necessary, is more time and cost-effective than conducting new reviews. Consequently, before embarking on a new systematic review and to avoid duplication, we undertook a rapid scoping review to assess the available synthesised research in this area.

### Objectives

The objectives of this rapid scoping review were to:

- Identify the number of existing systematic reviews on iodine for prenatal and postnatal growth;
- Assess the nature and scope of existing systematic reviews on iodine for prenatal and postnatal growth;
- Assess the methodological quality of existing systematic reviews on iodine for prenatal and postnatal growth;
- To identify any gaps in the evidence base for the effects of iodine for prenatal and postnatal growth and hence determine whether a new systematic review in this area is justified.

## Review

### Search strategy and selection criteria

To identify eligible systematic reviews we searched TRIP (http://www.tripdatabase.com) and Epistemonikos (http://www.epistemonikos.org). TRIP is a consistently updated clinical search engine with emphasis on evidence based medicine and clinical guidelines, including content from the Cochrane Database of Systematic Reviews (CDSR) and Pubmed. Epistemonikos is a collaborative, multilingual database of research evidence aiming to provide rapid access to systematic reviews in health that is maintained by systematically searching 25 databases for systematic reviews, broad synthesis or structured summaries, including CDSR, Pubmed and EMBASE.

Comprehensive electronic searches of TRIP and Epistemonikos were undertaken on 25^th^ March 2014 and updated on 15^th^ December 2014. In both instances, we used a simple, broad search string, being “iod*” and “systematic review” for TRIP and “iod*” for Epistemonikos. We searched for English language reviews, reporting on any iodine treatment or exposure that included experimental or observational studies in humans. All non-English and animal studies were excluded. Participants included were pregnant or lactating women or children up to the age of 18 years; adult populations were excluded. We selected studies that investigated exposure to iodised salt, iodised oil or iodine supplementation in any form, dose or regimen (including foods and iodine given in conjunction with other micronutrients) and included any adverse health outcomes due to iodine deficiency, including, but not limited to, effects on growth. No specific outcomes (e.g., cognitive development, weight-for-height z-scores) were pre-defined.

### Data collection and extraction

#### Selection of systematic reviews

Two authors (JF and LN) independently screened the titles and abstracts of all search results and identified potentially eligible systematic reviews using the pre-specified eligibility criteria. Where at least one author considered a study to be relevant, the full text document of the article was obtained. The first author in consultation with CN and LN did the screening of full text articles for final inclusion, and any uncertainties were resolved by discussion among authors. Reasons for excluding full-text articles were captured.

### Data analysis

Relevant information from the eligible systematic reviews was extracted and tabulated to capture the scope of the questions that were addressed by each review, including: author, year of publication, date of most recent search, types of participants, pre-specified treatment/exposure and comparator, health outcomes reported relevant to iodine deficiency and the number and types of studies included in the review. The validated and reliable AMSTAR tool [26, 27] was applied to each eligible systematic review to assess the methodological quality of the review. The maximum AMSTAR score is 11 for systematic reviews with meta-analyses, and 10 for systematic reviews without meta-analyses. Scores of 0 to 4 indicate low methodological quality, 5 to 8 moderate methodological quality, and 9 to 11 high methodological quality [28].

## Results

**Fig. 1:**
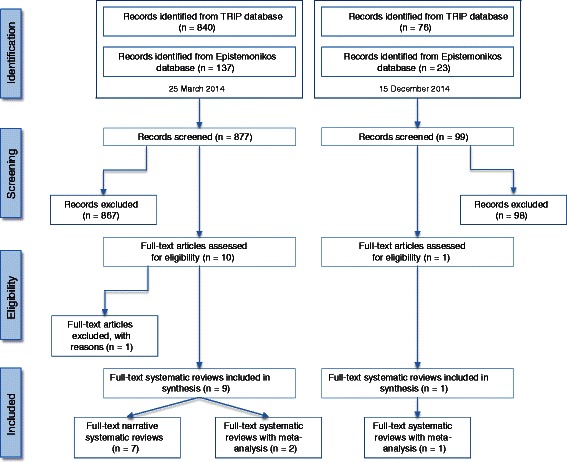
Flowchart of screening process for eligible reviews

The search results and selection process are detailed in Fig. 1. A broad search string was used in an attempt to ensure potentially eligible systematic reviews were not missed. Overall, the two searches yielded 976 records. After screening the titles and abstracts of all the records and removing the duplicates, we excluded 965 search results, as they were ineligible according to the pre-defined criteria. The majority of excluded studies covered irrelevant topics such as medical management of hyperthyroidism; nuclear medicine and radioiodine; skin antisepsis and wound care; use of iodine in dentistry; cardiovascular and diabetes care and cancer therapy. Other reasons for exclusion were incorrect participants (adults), non-English language publications and incorrect study design (not systematic reviews).

The full-text articles of eleven potentially eligible systematic reviews were obtained. One full text was excluded [29] as it is a duplicate of an included Cochrane Review [21]. Three of the ten eligible systematic reviews were Cochrane Reviews [21, 30, 31]. Seven of the eligible reviews used narrative synthesis and three included meta-analyses [8, 32, 33]. The detailed characteristics of the eligible systematic reviews and the gaps relevant to our research question identified in the existing reviews are described in Table 1.

**Table 1:** Characteristics of included studies

	Author & year	Aim of study	Participants; Study designs considered	Treatment / Exposure; Setting	Control, if applicable	Pre-defined outcomes	No. studies; Study designs included; Locations	Gaps relevant to our research question	AMSTAR score
	Last search date								
Quantitative studies (including meta-analyses)									
1	Taylor et al. (2014)	To evaluate the impact of iodine supplementation in pregnancy and childhood on thyroid function and child	School-age children from populations of mild-to-moderate iodine deficiency (determined from the median population urinary iodine)	Maternal iodine supplementation in pregnancy; Childhood iodine supplementation	No supplementation or significantly lower dose of supplements	Thyroid function; thyroid volume; cognitive performance	17 studies included in the review, of which 9 RCTs and 8 observational studies	Review only covers maternal and infant thyroid function, and child neurodevelopment.	8
	Last search date – April 2013 Ref.: [32]								
				Setting: Mild to moderate iodine deficiency			Relevant studies: 4 RCTs reporting on neonatal thyroid function	There are no growth outcomes considered.	
		Neurodevelopment in populations with mild-to moderate Iodine deficiency.	RCTs, quasi-randomised trials, prospective cohort or case–control studies considered						
							Locations: Belgium, Denmark, Germany and Spain	Not all relevant age groups are included (only neonates and school age children)	
2	Bougma et al. (2013)	To examine whether iodine status of mothers or infants affects the mental development of young children	Children 5 years and under RCT, non-randomised trial, prospective cohort trials considered	Exposure to different iodine levels before pregnancy, during pregnancy, or shortly after birth; or Examination of iodine exposure related to mental development outcome	Placebo, historical control, iodine sufficient siblings or children of similar age used as control group	Mental development score	24 studies included in the review, of which 2 RCT, 8 non-randomized intervention trials, 10 prospective cohort (women), and 9 prospective cohort (infants)	Review only investigates mental development.	8
	Last search date – November 2011 Ref.: [8]								
								There are no growth outcomes considered.	
								Not all relevant age groups are included (only under 5 years).	
							Relevant studies: None. No studies report on growth (total of 24 studies included in review)		
				Setting: Not defined					
							Locations: China, DR Congo, Ecuador, Peru, Spain, Portugal, USA, Netherlands, Italy, UK, Canada.		
3	Ristić-Medić et al. (2014)	To identify and examine studies investigating iodine intake and biomarkers of iodine status and to combine these studies in a meta-analysis to estimate the dose–response relationships between iodine intake and iodine status.	No criteria specified	For RCTs: Iodine intervention (iodised salt, iodised oil, iodised water, iodine tablets, iodine-enriched food or milk formula)	For RCTs: Placebo or low-dose iodine supplement (<100μg iodine per day)	For RCTs: Mean concentrations of UI, serum Tg, serum TSH, analytical methods to assess iodine status	58 studies included in the review, of which 33 RCTs 30 observational studies (5 being part of the included RCTs)	Review looked at iodine biomarkers. Does not consider iodine-related outcomes i.e., growth.	8
			RCTs, prospective cohort studies, nested case–control studies, cross sectional studies considered						
	Last search date – December 2011 Ref.: [33]								
						For observational studies: Concentration of UI, serum Tg, serum TSH, analytical methods to assess iodine status	Relevant studies: None		
				Observational studies: Evaluation of iodine intake (food frequency questionnaire, dietary history method, 24h recall, adherence to WHO criteria for assessing iodine intake)					
							Locations: Africa, Americas, Asia, Australasia, Europe		
				Setting: Not defined					
Qualitative studies (not including meta-analyses)									
1	Khor & Misra (2012) Last searched Ref.: [36]	To provide an update on the effects of micronutrient interventions (by supplementation or food fortification) on cognitive performance of children of 5-15 years in developing countries	Children 5-15 years RCTs only considered	Micronutrient (vitamins and/or minerals) supplementation for a period of >4 months	Not specified	Cognitive development indicators including: psychomotor development, cognitive performance, mental development, IQ, school performance.	13 RCTs included in the review, of which 6 that considered micronutrient-fortified foods including iodine.	Did not consider growth outcomes. Not all relevant age groups are included; 5 – 15 years only.	4
			Developing countries (UN classification)						
				(Studies that used other dietary components such as essential fatty acids, functional foods were excluded.)			Relevant studies: none	Only included RCTs.	
							Locations: Asia, Kenya, Morocco		
				Setting: Developing countries					
								Only UN-classification developing countries included	
2	Best et al. (2011) Last search date – not specified Ref.: [35]	To examine the impact of multi-micronutrient (MMN) food fortification on the micronutrient status, growth, health, and cognitive development of school-age children.	School age children. (defined by 75 % of study population being between 6 and 18 years)	MMN-fortified food (defined as food to which > 3 micronutrients were added)	Unfortified food or food fortified with only one or two micronutrients	Biochemical measurements of micronutrient status, prevalence of micronutrient deficiencies, indicators of growth or body composition, stunting, wasting, underweight, morbidities, absence from school, cognitive outcomes, academic performance.	12 studies included, of which: 6 controlled clinical trials (CCT), 1 controlled before-after (CBA) trial, and 5 RCTs	Looks at multiple MN rather than just supplementation with iodine alone.	6
			Experimental controlled efficacy or effectiveness studies including quasi-experimental controlled clinical trials, and controlled before-after studies only considered						
				Setting: Not defined			Relevant studies: 1 CCT compared effects of MMN fortification to single fortification with iodine (Morocco);4 studies reported on growth outcomes (height/stunting and weight/BMI/ underweight)	Fortified food only, and not supplementation.	
								Restricted to English language publications.	
								School age children only; review does not consider all relevant population groups.	
							Locations: Asia, Australasia, India, North Africa, Southern Africa		
3	Gunnars-dottir & Dahl (2012) Last search date – September 2010 Ref.: [34]	To assess the influence of different intakes of iodine at different life stages (infants, children, adolescents, adults, elderly, and during pregnancy and lactation), in order to estimate the requirement for adequate growth, development, and maintenance of health.	Not specified, except that publications must be either in English or a Nordic language; have >50 subjects; consider representative samples of the population or specific sub-samples of the population; have an indicator of iodine status and/or thyroid function, e.g.,: UIC, thyroid volume, TSH, T3 and T4	Not specified	Not specified	Pregnancy outcome, childhood development (including cognitive function and growth), thyroid function (thyroid hormones, thyroid gland size, hyper- and hypothyroidism), metabolism, health, and weight	40 studies of mixed design included, of which 2 studies (1 clinical trial, 1 cohort) reported on iodine status in pregnancy, pregnancy outcome and thyroid function of mother and infant 1 cross-sectional study reported on excess iodine intake and UIC	Included adults and elderly as well as children and pregnant women.	7
				Setting: Not defined					
								Some papers were excluded on the basis that they were not relevant to Nordic countries.	
								Does not only cover intervention studies- also included are observational studies.	
							Locations: Europe, Nordic countries, Americas, Eastern Mediterranean, Africa, Western Pacific		
								No specified intervention or control.	
			All study types considered						
4	Zhou et al. (2013) Last search date – December 2012 Ref.: [9]	To evaluate the efficacy and safety of iodine supplementation during pregnancy or the periconceptual period on the development and growth of children.	Pregnant or women of childbearing age, regardless of iodine status or gestation at trial entry	Any form of iodine supplementation, with or without other nutrients.	Absence of iodine between exposure groups.	Primary outcome: cognitive development of children	8 RCTs included in the review, of which 2 quasi-randomised	Only included RCTs.	8
								Not all relevant age groups are included – children of pregnant women are followed after pregnancy. Does not consider iodine supplementation in children themselves.	
			RCTs (including quasi-random design) only considered	Setting: Not defined		Secondary outcomes: pregnancy and birth outcomes, childhood growth and mortality, iodine status, thyroid function of mothers and infants.	Relevant studies: 2 quasi-RCT, reporting on growth outcomes (children at 5 years follow-up following intervention: skinfold thickness, MUAC, postnatal bone maturation growth rate; height of children at 15 years follow up; and pregnancy outcomes including birth anthropometrics and APGAR score)		
							Locations: Peru, Papua New Guinea (Q-RCTs); Belgium, Germany, Denmark, Italy, Chile.		
5	Ibrahim et al. (2006) Last search date – November 2005 Ref.: [31]	To determine whether dietary supplementation with iodine affects mortality and morbidity in preterm infants.	Preterm infants (less than 37 weeks completed gestation)	Iodine supplementation (> 30 μg/kg/day)	Placebo or no supplementation	Primary outcomes: Neonatal mortality and mortality prior to hospital discharge; neuro-developmental outcomes at ≥ 12-months; severe neurodevelopmental disability; cognitive and educational outcomes at age > 5 years	1 study included, which was an RCT.	Only preterm infants considered.	9
							Relevance of study: Primary outcome was thyroid hormones	Does not include pregnancy, nor older infants or children.	
			Controlled trials using random or quasi-random patient allocation considered	Setting: Not defined					
							Location: UK	Not focussed on growth, rather the prevention of mortality and adverse neurodevelopmental outcomes following preterm births.	
						Secondary outcomes: Severe respiratory distress syndrome; biochemical measures of thyroid function and iodine status			
6	Angermeyr & Clar (2004) Last search date - October 2003 Ref.: [30]	To assess the effects of iodine supplementation (e.g., iodised oil, salt, water, bread, supplements, tablets) in comparison with placebo or with each other on outcomes relating to iodine deficiency disorders in children	Children ≤ 18 years living in areas with low iodine intake (iodine deficiency)	Any population-based iodine supplementation (e.g., iodised salt, iodised oil (given orally or by injection), iodised water, iodine tablets iodine added to food etc.)	Placebo or other iodine supplementation	Primary outcomes: Goitre rate and thyroid size	Total 26 studies included, of which 15 RCTs, 5 non-randomised controlled trials, 3 prospective controlled studies, 2 quasi-randomised trials, 1 prospective comparative study.	Review does not cover pregnancy.	9
						Physical development (height, weight, strength); mental development (measurement of cognitive function)			
							Relevant studies:22 studies measured one or more thyroid outcomes (goitre rate, thyroid size, urinary iodine excretion, THS, T3, T4, thyroglobulin).		
				Setting: Low iodine intake		Secondary outcomes: Mortality related to iodine deficiency disorders; symptoms and signs of hypothyroidism; urinary iodine concentration; blood TSH concentration; serum thyroglobulin concentration; adverse effects (e.g., iodine-induced hyperthyroidism, thyroid auto-antibodies); health-related quality of life; acceptability of supplement; compliance, costs; socioeconomic effects (e.g., school performance)			
							6 studies measured one or more growth outcome measures (height, weight, mortality)		
							Locations: North Africa, Asia Europe, India, Africa, Americas		
			RCT, quasi-randomised trials and prospective non-randomised experimental studies considered						
7	Wu et al. (2002) (updated 2004) Last search date – August 2004 Ref.: [21]	To assess the effects of iodised salt in comparison with placebo and other forms of iodine supplementation on the incidence of iodine deficiency disorders	Adults and children living in areas of low iodine intake	Iodised salt	Placebo, other forms of iodine supplementation (iodised oil, iodised water, etc.)	Primary outcomes: Mortality related to iodine deficiency disorders, goitre, physical and mental development in children, symptoms of hypothyroidism.	6 studies included, of which 1 RCT, 3 RCT (not blinded, blinding unknown, participants unblinded outcome assessment blinded), 2 prospective controlled study	Only covers iodised salt.	9
				Setting: Low iodine intake					
			Any prospective study with a control group considered					All study types (with control group) considered	
						Secondary outcomes: UIC, TSH in blood and neonatal cord blood, serum thyroglobulin, adverse effects (e.g., iodine induced hyperthyroidism), health related quality of life, costs, compliance, socioeconomic effects		Also considers adults	
							Relevant studies: 1 RCT reported on UIC		
							Locations: Germany, China, South Africa, Italy, Malaysia, India		

### Scope of included studies

#### Interventions

Eight of the ten systematic reviews look at some form of iodine intake, specifying either supplementation in various forms (salt, iodised oil, iodine supplements, food fortified with iodine e.g., bread) [9, 29–32], or exposure to iodine (i.e., indication of iodine status or thyroid function) [8, 33, 34]. Two systematic reviews consider multiple micronutrients: as food fortification [35], or micronutrient interventions by supplementation or fortification [36].

Of the five reviews specifying supplementation of iodine intake, one systematic review looks at all forms of population-based iodine supplementation, e.g., iodised salt, iodised oil (orally or by injection), iodised water, iodine tablets, food fortified with iodine (but excluding multiple micronutrients or food fortification), in comparison to placebo, non-iodised control or each other [30]. Another review focused on any prospective study investigating the prevention of iodine deficiency disorders using iodised salt, which had a control group (placebo or other forms of iodine supplementation e.g., iodised oil, iodised water) [21]. Three further systematic reviews investigate direct iodine supplementation (any form of supplementation [9, 32]; and supplementation appropriate to premature infants, to achieve an intake of more than 30 μg per kilogram bodyweight per day [31]).

#### Setting

Three systematic reviews specify particular settings with respect to iodine intake: one review focuses explicitly on mild to moderate iodine deficiency settings [32], and two reviews stipulate settings of low iodine intake [21, 30]. A further systematic review is focused on developing countries only [36], however, the remaining six papers did not specify iodine deficient, replete or excess settings. Of these six systematic reviews, one systematic review aims to estimate the dose–response relationship between iodine intake and iodine status, but does not address health-related outcomes [33]. It includes studies from severe, moderate and mild iodine-deficient settings, and replete settings. Studies investigating excessive iodine intake were also eligible, however, no such studies were found.

#### Study design

Most of the reviews include randomised controlled trials (RCT) as well as other study designs such as non-randomised trials, quasi-RCTs, prospective comparative studies and cohort studies. Two reviews include RCTs only [9, 36]. In the majority of the narratively synthesised reviews, the included studies had control groups and authors attempt to present a comparative narrative appraisal when a meta-analysis was not done or not possible. Three reviews feature meta-analyses [8, 32, 33]. Conversely, Gunnarsdottir and Dahl [34] examine the literature available at time of writing, with the objective of reviewing and updating the fourth (2004) edition of the Nordic Nutrition Recommendations. As such, this is different to the other included reviews in that it does not have specific inclusion criteria for study type or population nor control group, requiring only that studies are representative of the Nordic population and comment on one or more indicators of iodine status.

#### Population groups

The systematic reviews included in this rapid scoping exercise cover populations of pregnant women and women of reproductive age through to subjects of age 18, and three reviews also include adults in addition to children [21, 33, 34]. Only one study looks at the periconceptual period and pregnancy [9], including all women of reproductive age. Ibrahim and colleagues review only preterm infants of less than 37 weeks gestation [31]. One review specifies children under 5 years [8], three focus on school-age children [32, 35, 36], and one considers minors until age 18 [30]. One systematic review does not look at specific population groups, since its aim is instead to examine studies investigating iodine intake in relation to biomarkers of iodine status [33]. Here, no population group is excluded, except patient populations.

#### Control groups

Controls used in the included systematic reviews are placebo [8, 21, 30, 31, 33], no supplementation of iodine [31, 32], absence of iodine between exposure groups [9] unfortified foods or foods fortified with only one or two micronutrients [35], low doses of supplements [32, 33], other iodine supplements [21, 30], historical controls [8] and iodine sufficient siblings used as a control [8]. Two systematic reviews did not specify control groups [34, 36].

#### Outcomes

Table 2 provides a summary of the outcomes defined within each systematic review. As can be seen from the table, only half of the included systematic reviews include pre-defined growth outcomes [9, 21, 30, 34, 35]. Amongst the remaining reviews, three address cognitive outcomes [8, 32, 36] (a fourth review, discussed below, cites cognitive development as a primary outcome with growth as a secondary outcome [9]). One review looks specifically at premature infant morbidity and mortality [31]. Lastly, Ristić-Medić et al. [33] investigate biomarkers of iodine.

**Table 2:** Summary of pre-defined outcomes investigated in included systematic reviews

Systematic review refe	Growth indicators (weight, height, stunting, wasting, strength)	Pregnancy/birth outcomes	Iodine status (UIC, goitre rate)	Thyroid function (thyroid hormones (Tg, TSH), thyroid volume, hyper- & hypothyroidism)	Analytical methods to assess iodine status	Cognitive outcomes (performance, mental development score, psychomotor development)	Academic performance, IQ, school absence	Health related quality of life, socioeconomic effects	Health related quality of life, socioeconomic effects Compliance with supplement/fortification, acceptability, costs	Adverse events/morbidity, mortality	Prevalence of micronutrient deficiencies
Taylor et al., 2014 [32]	✗	✗	✗	✓	✗	✓	✗	✗	✗	✗	✗
Bougma et al., 2013 [8]	✗	✗	✗	✗	✗	✓	✗	✗	✗	✗	✗
Ristić-Medić et al., 2014 [33]	✗	✗	✗	✓	✓	✗	✗	✗	✗	✗	✗
Khor & Misra, 2012 [36]	✗	✗	✗	✗	✗	✓	✓	✗	✗	✗	✗
Best et al., 2011 [35]	✓	✗	✗	✗	✗	✓	✓	✗	✗	✓	✓
Gunnarsdottir & Dahl, 2012 [34]	✓	✓	✗	✓	✗	✓	✗	✗	✗	✗	✗
Zhou et al., 2013 [9]	✓	✗	✓	✓	✗	✓	✗	✗	✗	✓	✗
Ibrahim et al., 2006 [31]	✗	✗	✓	✓	✗	✓	✗	✗	✗	✓	✗
Angermeyr & Clar, 2004 [30]	✓	✗	✓	✓	✗	✓	✗	✓	✓	✓	✗
Wu et al., 2002 [21]	✓	✗	✓	✓	✗	✓	✗	✓	✓	✓	✗

##### Studies including growth-related outcomes

Of the five reviews including growth outcomes, none conducted meta-analyses. Growth outcomes in these reviews are included as either primary and secondary outcomes, but generally, outcomes are not explicitly defined, with authors preferring to describe outcomes under the umbrellas of “childhood growth” [9], “physical development” [21, 30] and “childhood development (including growth)” [34]. That said, one systematic review states precise measures of growth, namely “prevalence of stunting, wasting or underweight” in addition to a more general outcome of “indicators of growth or body composition” [35]; this study investigated the effects of multiple micronutrients on the growth, health and cognition of school children, so inclusion of such terms is expected. Upon examination of the exact search terms used in these systematic reviews, two included a search term for growth (“physical, body growth” [9], and “growth and development” [34]), one review used “development” only [21], and one review used no growth or development terms at all [30]. (The final review of the five described did not publish their search terms [35].) One review looked specifically at the effects of iodine supplementation during pregnancy or the periconceptual period on later child development [9], the other four reviews considered postnatal growth, including school-age children [35], children to age 18 years [30], and all ages including adults [21, 34].

Despite the intentions of these reviews, evidence on effects of iodine supplementation or fortification on somatic growth outcomes was sparse. Best et al. [35] report on four studies which showed a significant effect on weight and body mass index (BMI) in school children receiving fortified foods, however this was achieved with multiple micronutrient interventions and improved outcomes could not be attributed to iodine alone. Angemeyr and Clar [30], investigating iodine supplementation in any form on children under 18 years, found one study where significant differences were found for two measures of physical stamina, however the other four studies which reported on growth did not see significant differences in physical development during the time periods investigated. Most studies included in this review assessed the use of iodised oil with only a few looking at other forms of iodine supplementation. This review thus offers little evidence on physical development from all forms of iodine supplementation. Gunnarsdottir and Dahl [34] in their broad literature review found only cross-sectional studies reporting on growth. Zhou et al. [9] consider iodine supplementation in pregnancy, and discuss at length two older studies (>40 years) conducted in regions of severe iodine deficiency where growth was investigated, however, no other trials reporting growth outcomes were included. Wu et al. [21] do not discuss any growth outcomes in their results.

### Methodological quality

**Fig. 2:**
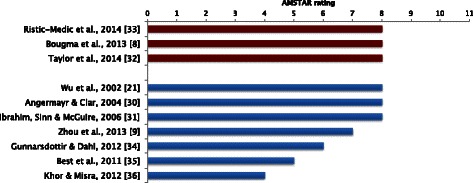
AMSTAR rating of studies. Note: denominator of 11 for systematic reviews including a meta-analysis; denominator of 10 for narrative systematic reviews. Red colour indicates meta-analyses; blue indicates narrative systematic reviews

The total AMSTAR scores for the included papers are shown in Fig. 2. No reviews were rated as “high” methodological quality, a score of 9 or more. Most reviews were rated as “moderate”, i.e., scoring between 5 and 8 [8, 9, 21, 30–35], and one review achieved a low score (4 or less) [36].

**Fig. 3:**
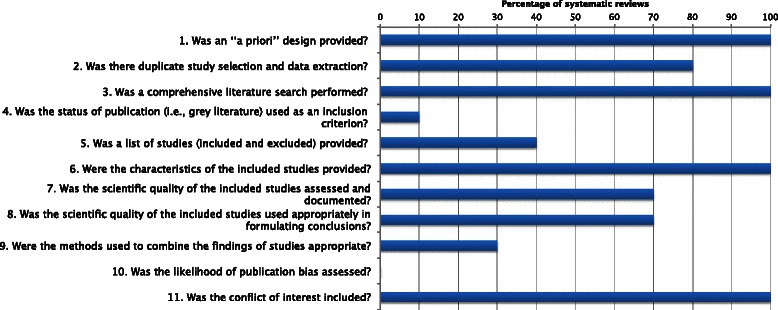
Breakdown of AMSTAR score per domain

The chart in Fig. 3 details the scoring in each domain. The most common “problem domains” (domains 4, 5, 7, 8, 10; Fig. 3), where methodological quality points were not gained are discussed below. Only one review included explicit statements about publication status as an inclusion criterion [31]. Ideally systematic reviews should include both published and unpublished studies or “grey literature” [37], and authors should state that searches were conducted regardless of study publication type and whether studies were excluded based on publication status or other factors such as language. A list of included and excluded studies is also not provided in many of the eligible systematic reviews [8, 9, 32, 33, 35, 36]. The AMSTAR tool also states that methods of assessing scientific quality of studies should be stated *a priori*, and that the scientific quality of data should be taken into account when reaching conclusions and explicitly stated when making recommendations based upon study results [26]. An assessment of the scientific quality of included studies is missing in one systematic review [36], and two further reviews fail to state the methods of assessment *a priori* [31, 35]. Of the other systematic reviews, three state the intent to use Cochrane tools (Cochrane Risk of Bias tool; Cochrane Handbook) in assessing the scientific quality of included literature [9, 21, 33], three reviews [21, 30, 32] also discuss using a modification of previously published quality criteria recommendations by Jadad [38] and/or Schultz [39], two reviews describe the use of other tools [32, 34], and one review does not specify the use of a particular tool to assess the scientific quality of included literature, however describes the methodology intended to be used [8]. Three reviews fail to integrate the scientific quality of the included studies into the certainty of the review findings and conclusions [34–36]. Lastly, none of the systematic reviews assessed the likelihood of publication bias, a known threat to the validity of systematic reviews [27]. The AMSTAR developers Shea et al. [26, 27] state that assessments of publication bias should include a combination of graphical aids e.g., funnel plots and/or statistical tests [8, 32, 33].

## Discussion

The objective of this rapid scoping review was to identify the scope, methodological quality and nature of existing systematic reviews that have investigated the effects of iodine supplementation or fortification on prenatal and postnatal growth in children and adolescents. This scoping review has identified gaps in the evidence base related to this this question and has facilitated and informed the development of a protocol for a new systematic review that will specifically seek to address these gaps [40].

We identified ten systematic reviews that investigate health-related issues associated with iodine intake. Aspects covered include thyroid function, iodine deficiency disorders, pregnancy outcomes, cognitive development, and growth. One review also looks at iodine biomarkers and the relationship to iodine intake. Most reviews evaluate iodine supplementation in one or many forms. Participants are mainly school-age children, though most reviews also include other population groups, i.e., adults, pregnant women and preterm infants. Most comparison groups entail a placebo or no iodine supplementation.

Methodological quality of the included systematic reviews can be deemed as fair, with nine out of the ten reviews scoring a “moderate” rating using the AMSTAR tool. One review was rated as having low methodological quality [36]. AMSTAR domains where systematic reviews did not score well were the use of publication status as an inclusion criterion, and the evaluation of publication bias. These points are particularly important, since publication bias is a well-described threat to systematic review validity [27], and the inclusion of all eligible studies in systematic reviews and meta-analyses is required to reduce this risk of bias [37].

Five of the ten systematic reviews included in this rapid scoping review include growth outcomes within the scope of their literature examination [9, 21, 30, 34, 35]. Only one narrative systematic review published in 2004, assesses the effect of improved iodine nutrition on the physical development of children below the age of 18, via any form of population-based iodine supplementation or fortification strategy [30]. However, this review does not offer comprehensive, up-to-date synthesised evidence on the effects of iodine nutrition on growth. Furthermore, prenatal growth outcomes or supplementation during pregnancy are not included, and the review does not incorporate any specific growth outcomes in its search terms, hence some studies with a focus on growth may have been missed. Lastly, this review is over ten years old, and will thus not consider the most recent literature. The further four reviews which include growth outcomes give only little discussion on growth, if any, and again, do not cover all relevant population groups. Two of these systematic reviews specify populations with low iodine intake [21, 30], and a further review reports on studies conducted in regions of severe deficiency [9]. Only one systematic review included in this scoping review attempts to clarify the dose–response relationship between iodine status and iodine intake [33], but despite the inclusion of thyroid function markers, this review was not focused on health outcomes, and thus could not clarify to what point iodine deficiency may affect growth (i.e., at mild deficiency, or just moderately or severely deficient settings). Overall, these results point to a lack of synthesised and up-to-date evidence on the effects of iodine on somatic growth from the prenatal period up to age 18 years.

## Conclusion

This scoping review identifies a gap in the current evidence base on iodine for growth. None of the identified systematic reviews investigate the effects of all forms of iodine supplementation/fortification in all of the relevant population groups (i.e., women of childbearing age, pregnant and lactating women and children of all ages) on all of the relevant growth and growth-related outcomes. A good quality systematic review of studies investigating the effects of all forms of iodine supplementation and fortification in all relevant population groups on prenatal and postnatal somatic growth is needed to address this gap, and would provide important evidence on strategies to prevent stunting. This rapid scoping review has informed and supported the development and publication of a protocol for a new systematic review [40] that will examine the effects of all forms of iodine supplementation and fortification on somatic growth throughout the life stages of the child. The results of this systematic review have the potential to contribute to and enhance the evidence base that can inform decisions regarding iodine supplementation/fortification and child growth.

## Abbreviations

BMI: Body Mass Index
IDD: Iodine Deficiency Disorders
RCT: Randomised Controlled Trial
UNICEF: United Nations Children’s Fund
WFP: United Nations World Food Programme
WHO: World Health Organization
